# Findings and Outcome of Transcatheter Right Ventricular Endomyocardial Biopsy and Hemodynamic Assessment in Children with Suspected Myocarditis or Cardiomyopathy

**DOI:** 10.3390/ijerph191610406

**Published:** 2022-08-21

**Authors:** Alessia Callegari, Daniel Quandt, Achim Schmitz, Karin Klingel, Christian Balmer, Hitendu Dave, Oliver Kretschmar, Walter Knirsch

**Affiliations:** 1 Pediatric Cardiology, Pediatric Heart Center, Department of Surgery, University Children’s Hospital Zurich, 8032 Zurich, Switzerland; 2 Children’s Research Centre, University Children’s Hospital Zurich, 8032 Zurich, Switzerland; 3 University of Zurich, 8006 Zurich, Switzerland; 4 Division of Anesthesia, University Children’s Hospital Zurich, 8032 Zurich, Switzerland; 5 Cardiopathology, Institute for Pathology, Eberhard Karls University Tübingen, 72074 Tubingen, Germany; 6 Congenital Cardiovascular Surgery, Pediatric Heart Center, Department of Surgery, University Children’s Hospital Zurich, 8032 Zurich, Switzerland

**Keywords:** right ventricular endomyocardial biopsy, children, cardiomyopathy, myocarditis

## Abstract

Objective: The study objective is assessing findings and outcome in children with suspected cardiomyopathy (CMP) or myocarditis undergoing cardiac catheterization with transcatheter right ventricular endomyocardial biopsy (RV-EMB). Methods: All consecutive children undergoing cardiac catheterization with RV-EMB for suspected CMP/myocarditis between 2002–2021 were analysed regarding clinical presentation, cardiac biomarkers, periprocedural management, hemodynamic, histological/immunohistological findings, and outcome. Results: Eighty-five RV-EMBs were performed in 81 patients at a median age of 6.8 (IQR 9.9) years and a bodyweight of 20 (32.2) kg. Histological/immunohistological findings of RV-EMB revealed dilated CMP in 10 (12%), chronic myocarditis in 28 (33%), healing myocarditis in 5 (6%), acute myocarditis in 9 (11%), other heart muscle diseases in 23 (27%) (7 restrictive CMP, 5 hypertrophic CMP, 4 toxic/anthracycline-induced CMP, 4 endocardfibroelastosis, 1 arrhythmogenic right ventricular CMP, 1 laminin CMP, 1 haemangioma), no conclusive histology in 7 (8%), and normal histology in 3 (4%) patients. Median LVEDP was 17 mmHg (IQR 9), LAP 15 mmHg (10), and PVR 1.83 (1.87) Wood Units/m^2^. There were 3 major complications (3%), all patients recovered without any sequelae. At follow-up (median 1153, IQR 1799 days) 47 (59%) patients were alive, 11 (13%) dead, 15 (18%) underwent cardiac transplantation, and 8 (9%) were lost to follow-up. Death/cardiac transplantation occurred within 3 years from RV-EMB. All patients with an acute myocarditis survived. NT-pro-BNP, echo parameters, and invasive hemodynamics correlate independently with death/cardiac transplant. Conclusion: Hemodynamic invasive data and morphological findings in RV-EMB complete clinical diagnosis in children with suspected CMP/myocarditis and provide important information for further clinical management.

## 1. Introduction

Reduced systolic or/and diastolic myocardial function may be caused by myocardial inflammation due to acute or chronic, mostly viral myocarditis or due to acute cardiac decompensation in children with primary/former cardiomyopathy (CMP) [1,2,3,4,5,6]. These different clinical entities may cause significant long-term morbidity, with potential adverse outcomes including terminal heart failure, need for heart transplantation, and increased risk for sudden cardiac death [1,7,8,9,10]. 

Acute myocarditis can show a very heterogenous clinical presentation from asymptomatic solitary electrocardiographic abnormalities, through fulminant acute heart failure to sudden cardiac death [1,3,5], and may resolve into a chronic inflammatory process [6]. Therefore, diagnosis and clinical management remain difficult and partly differ between children and adults [11]. Guidelines on its management include laboratory testing, electrocardiography and echocardiography, cardiac magnetic resonance (cMRI), and right ventricular endomyocardial biopsy (RV-EMB) [3,4,12,13]. RV-EMB are analysed with defined histological and immunohistological criteria as well as molecular findings including PCR and in situ hybridization for viral genome detection. Over the last decade, diagnosis of CMP or myocarditis has shifted from being mainly biopsy-based to being also cMRI-based in many scenarios [3,4]. However, RV-EMB still delivers information about the etiopathogenesis of the (inflammatory) heart disease that can become essential for an optimal individual patient management. This includes early diagnosis and specific care, including circulatory support measures with inotropes and mechanical devices and eventual immunomodulatory or immunosuppressive therapy [3,4,14,15].

Nevertheless, especially in children, RV-EMB are not routinely performed in many centres due to concerns regarding the procedural risks (including complications such as arrhythmias, myocardial perforation, pericardial tamponade, myocardial failure, and death) [16,17]. Data regarding peri-procedural safety of RV-EMB in most severely affected children are rare [18]. Furthermore, clinical impact of hemodynamic data and histological/immunohistological findings of RV-EMB including specific viral diagnostics on further clinical management and outcome are limited [3,4,19]. Lastly, predictors of poor outcome remain unclear and controversial [20]. 

Therefore, aim of this study was to evaluate hemodynamic and histological findings of cardiac catheterization with RV-EMB as standard of care in children treated for myocarditis/CMP and their potential relation to later clinical outcome.

## 2. Materials and Methods

### 2.1. Study Design

This is a single centre, retrospective, longitudinal analysis of a paediatric population with suspected viral myocarditis or CMP between 2002 and 2021 (20 years). We included all consecutive patients undergoing cardiac catheterization with RV-EMB performed for diagnostic work-up due to newly diagnosed, severe systolic or diastolic myocardial left ventricular dysfunction associated with ventricular dilatation, hypertrophy, or restriction. Clinical presentation and findings on two-dimensional transthoracic echocardiography were suspicious of myocarditis, CMP (dilated, restrictive or hypertrophic) or systemic ventricle failure for unknown reasons. Purpose of RV-EMB was to confirm or rule out the diagnosis of myocarditis or CMP. 

### 2.2. Cardiac Catheterisation

Eighty-two (96%) transcatheter RV-EMBs were performed antegradely (using venous access and antegrade right heart catheterization). In three patients (4%) with single ventricle physiology a retrograde arterial approach was used. Site of RV-EMB was the apical or septal region of the right ventricle (RV) in all procedures and at least 6 separate biopsies (3× formalin, 3× RNAlater^®^) were taken with a Cordis^®^ Biopsy forceps 5.5 French (Ref. Nummer 504-300). A coronary artery angiography ruled out coronary abnormalities such as abnormal left coronary artery originating from the pulmonary artery, coronary artery fistula, or myocardial bridging in all patients. In case of coronary abnormalities, no RV-EMB was performed.

### 2.3. Clinical Data

The five main categories for further analysis were (1) clinical presentation and biomarkers (NT-pro-BNP starting from 2010), (2) hemodynamic findings of cardiac catheterization, (3) peri-procedural course of cardiac catheterization, (4) hemodynamic findings of cardiac catheterization, (5) histological/immunohistological findings of RV-EMB, and (6) outcome. Analysis of peri-procedural course included anaesthetic data such as new establishment of inotropic support (milrinone/adrenaline/noradrenaline) during cardiac catheterization and catheter-related complications. Further analysis included review of all available hemodynamic data including left ventricular end-diastolic pressure (LVEDP), left atrial pressure (LAP) or pulmonary capillary wedge pressure, and mean pulmonary artery pressure (mPAP). The outcome and survival of this cohort was assessed over a period of 658 (IQR 1337.5) days. Z-scores for echocardiographic parameters were taken from Pettersen et al. [21].

### 2.4. Histological/Immunohistological Analyses

Myocardial inflammation for histological analysis of paraffin-embedded RV-EMB was defined as the detection of 14 infiltrating leukocytes/mm² (CD3+ T-lymphocytes and/or CD68+ macrophages) in addition to enhanced human leukocyte antigen II expression in antigen-presenting cells as described [12,22]. Acute myocarditis revealing myocyte necrosis in the presence of severe inflammation was differentiated from healing/chronic myocarditis, which was defined by the following criteria: absence of acute myocyte necrosis but detection of interstitial fibrosis, ≥14 infiltrating leukocytes/mm² (CD3+ T-lymphocytes and/or CD68+ macrophages) and degeneration of neighbouring myocytes, being morphologically consistent with the formerly defined “borderline” myocarditis [23,24]. For the detection of viral genomes nested (RT-) polymerase chain reaction was performed in RV-EMB fixed in RNAlater^®^ [13,25]. Quantitative parvovirus B19 PCR was done as described [26]. Molecular biological detection of nucleic acids form cardiotropic viruses performed by nested (RT-) PCR were also performed from peripheral lymphocytes.

### 2.5. Statistics and Ethics

Statistical analysis was performed using SPSS 25.0.0 (SPSS Inc., IBM Company, Chicago, IL, USA). Continuous variables are expressed as median (IQR), categorical data as counts and percentages. Group comparison was performed using two-sample *t*-tests and Levene’s test for equality of variance was used to analyse if the variability in the two groups was significantly different. Ordinal, nominal, and dichotomic variables were evaluated with contingency tables and compared with chi-square-tests or Kolmogorov–Smirnov analyses. Predictability of the continuous variables was evaluated by means of Pearson correlations and stepwise multiple regressions, with the criteria probability of F to enter *p* < 0.05, probability of F to be removed *p* > 0.1. Survival analysis was performed with Kaplan–Meier analyses (Log-rank tests) and Cox stepwise linear regressions. As values of NT-pro-BNP were markedly skewed, a logarithmic transformation was performed and used in all statistical tests involving this parameter. Significance is defined by values of *p* < 0.05.

The study protocol was approved by the local ethics authorities and adheres to the declaration of Helsinki (version 2013). 

## 3. Results

### 3.1. Clinical, Echocardiographic Findings and Cardiac Biomarker at Hospital Admission

Eighty-one patients (37 male) at a median age of 6.8 (IQR 9.9, min 3 weeks, max 17.1 years) years, bodyweight of 20.0 (32.2) kg, and body surface area (BSA) of 0.8 (0.8) at time of catheterization were included (Table 1). The time interval between hospital admission and RV-EMB was in median 5 (IQR 8) days. Prior to RV-EMB the patients were stabilized at our ICU, if necessary.

Suspected clinical diagnosis before cardiac catheterization and RV-EMB was dilated CMP in 46 (54%); restrictive CMP in 10 (12%); hypertrophic CMP in 4 (5%); viral myocarditis in 17 (20%); systemic ventricle failure for unknown reason (such as ventricular failure in patients with univentricular heart disease after Fontan-procedure or ventricular failure due to arrhythmias) in 4 (5%) patients. Most frequent clinical symptoms were fatigue in 55 (65%); tachypnoea in 46 (54%); presence of cardiac murmur in 43 (51%); hepatomegaly in 39 (46%); and tachycardia in 34 (40%) patients. A total of 18 (21%) patients presented with cardiopulmonary decompensation. Echocardiography showed a median (IQR) LV-ejection fraction (EF) of 35% (28); LV-SF of 20% (16.5); LVEDD z-score of +2.61 (3.73); and LVES z-score of +4.30 (4.83). 

Maximal Troponin (*n* = 61) was 4.0 [27] times elevated from the expected normal value. NT-pro-BNP (*n* = 56) was 18.5 (63.2) times elevated from the expected normal value. 

A total of 33 (42.8%) patients were treated with intravenous immunoglobulins and 11 (14.2%) with steroids prior to RV-EMB.

### 3.2. Periprocedural Management

Cardiac catheterizations were performed under general anaesthesia with endotracheal intubation. Physical patients’ status was classified per American Society of Anaesthesiologist physical status classification system and median score was 3 (2–4). Overall, inotropic support was necessary in 42 (50%) patients. On referral to the cardiac catheterization laboratory 28 patients (34%) were already on inotropic support. Out of these 28 patients inotropic support was maintained stable throughout the procedure in 18 patients (22%), whereas in 10 patients (12%) the dose needed to be increased or an additional inotropic medication had to be added. 

In 11 patients (7 with newly established continuous inotropic support and 4 with necessity to increase inotropic support) additional treatment was necessary. 

Adjustments of medication were performed due to low cardiac output and to maintain sufficient systemic arterial blood pressure throughout the procedure. In three cases inotropic support was increased due to periprocedural complications (see paragraph below). Younger age (*p* = 0.8), weight (*p* = 0.67) and length (*p* = 0.99), and cardiac biomarkers (NT-pro-BNP *p* = 0.23; Troponin *p* = 0.53) did not correlate to a higher need of inotropic support. 

Under this treatment peri-procedural blood gas investigations revealed lactate levels of median 1.3 (1.3), pH of 7.40 (0.1), and base excess of −2 (−2.6).

Complete hemodynamic assessment (Table 2) during cardiac catheterization including endomyocardial biopsy could be performed in 83 cases. Procedure time of cardiac catheterization was in 62 min (32.5) with an X-ray time of 12 min (6.7). Hemodynamic assessment revealed elevated LVEDP 17 mmHg [8] and LAP 15 mmHg [9]. Pulmonary hypertension with mPAP ≥ 25 mmHg was found in 33 (34%) hemodynamic assessments, PVR was 1.83 (1.87) Wood Units/m^2^ and central venous oxygen saturation (ScvO2) 70 (13) %. 

Periprocedural complications were major (*n* = 3, 3%) including right ventricular perforation during RV-EMB with immediate surgical suture needed (*n* = 2) and temporary complete AV block (*n* = 1), and minor (*n* = 6, 7%) including arrhythmia or femoral vessel thrombosis. Periprocedural complications occurred more often in patients with a higher LVEDP (*p* = 0.03) and lower central venous oxygen saturation (ScvO2) (*p* = 0.006). No differences regarding length of hospitalization prior to RV-EMB, age, weight and length, echocardiographic parameters, RV-EMB results, inotropic support, or cardiac biomarkers were found between the patients with and without complications. All patients recovered without further sequalae. 

### 3.3. Histological Findings

Four patients received a second RV-EMB during follow-up. The second biopsy was performed to monitor the effect of the established therapy in case of failing clinical improvement. Therefore, a total of 85 RV-EMB specimen were analysed. Histological/immunohistological findings revealed myocarditis in 42 of 85 (49%) patients (9 acute, 5 healing, and 28 chronic myocarditis). Dilated cardiomyopathy was histologically diagnosed in 10 cases (12%). Other types of heart diseases were identified in 23 cases (7 restrictive cardiomyopathy, 5 hypertrophic cardiomyopathy, 4 anthracycline-induced CMP, 4 endocardfibroelastosis, 1 arrhythmogenic right ventricular cardiomyopathy, 1 laminin cardiomyopathy, and 1 haemangioma). Unspecific mild changes (e.g., interstitial fibrosis, unspecific myocardial lymphocyte infiltration) without any strong evidence for myocarditis were found in seven (8%) patients. Normal RV-EMB results were found in three (3%) patients.

Molecular biological detection of nucleic acids form cardiotropic viruses performed by nested PCR from RV-EMB and peripheral lymphocytes are shown in Table 3. Human herpesvirus type 6 (HHV6) and parvovirus B 19 (PVB19) were predominantly detected in the myocardium, often concomitant, while HHV6 and HHV7 were common in lymphocytes. In nine RV-EMBs the same pathogen was found in the myocardium as well as in the lymphocytes suggesting acute systemic virus infection. The relationship between histological findings of myocarditis and viral RNA/DNA detection is shown in Table 3.

### 3.4. Diagnostic and Therapeutical Impact of Right Ventricular Endomyocardial Biopsy

The major impact of RV-EMB includes definite histological diagnosis in 71 (84%) patients. Although initially dilated CMP of unknown aetiology was suspected clinically in 42 patients, only 10 patients had histologically proven dilated CMP. Restrictive CMP was suspected in 10 patients and the diagnosis was confirmed in seven cases. Viral myocarditis was clinically suspected in 17 patients but in RV-EMB 42/85 (49%) patients had signs of acute or healing or chronic myocarditis. There were nine patients with histological signs of acute myocarditis and five patients were positive for RNA/DNA of cardiotropic viruses in RV-EMB. 

In 77 patients, the clinical course after RV-EMB was assessed. Diagnostic impact of RV-EMB resulted in a further treatment with intravenous immunoglobulins in 13 patients (17%), steroids in 5 (6%), redo of RV-EMB in 4 (5%), and immunosuppressive treatment with cyclosporine in 1 (1%). This last patient with cyclosporine treatment was critically ill with severe hemodynamic impairment, reduced ventricular function and had histological signs of healing myocarditis with positive viral load (cytomegalovirus) in both, blood lymphocytes and the myocardium and later died on the paediatric intensive care unit. 

Nine patients received surgical treatment following RV-EMB and CC results: cardiac tumour resection was performed once; six patients underwent mitral valve annuloplasty; and two surgeries for congenital heart disease (VSD-closure and ASD-closure). 

A cardioverter-defibrillator (ICD) was implanted in 16 (21%) patients after a median of 48 (IQR 148) days from RV-EMB. 

Twenty-three (30%) patients needed temporary invasive mechanical assist device support (LVAD in 16 patients, ECMO in 7) at a median interval of 24 days (IQR 66) from RV-EMB. In 8 patients this was as a “bridge-to-recovery” (2 patients with DCMP, 5 with myocarditis, 1 with non-conclusive histology), whilst 15 patients later underwent cardiac transplantation. In total 23 (30%) of the patients were listed for cardiac transplantation following their invasive assessment of hemodynamics and RV-EMB. 

### 3.5. Outcome and Outcome’s Predictors

At follow-up (median 1153, IQR 1799 days) 47 (59%) patients were alive (without cardiac transplantation), 11 (13%) patients died, 15 (18%) underwent cardiac transplantation, and 8 (9%) were lost to follow-up. 

**Figure 1:**
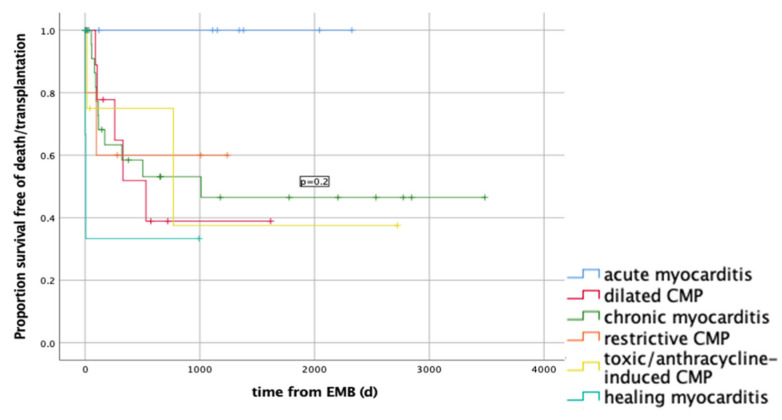
Survival function in relation to the most frequent histological findings (*p* = 0.2). Death/transplantation occurred in 0/9 (0%) patients with acute myocarditis; 5/10 (50%) with a dilated CMP; 11/25 (44%) with a chronic myocarditis; 2/3 (60%) with a restrictive CMP; 2/4 (50%) with a toxic/anthracycline-induced CMP; 2/4 (50%) with a healing myocarditis (50%).

Survival function in relation to the most frequent histological findings (*p* = 0.9) is shown in Figure 1. Death/transplantation occurred in 0/9 (0%) patients with acute myocarditis; 5/10 (50%) with a dilated CMP; 11/25 (44%) with a chronic myocarditis; 2/3 (60%) with a restrictive CMP; 2/4 (50%) with a toxic/anthracycline-induced CMP; 2/4 (50%) with a healing myocarditis (50%). Death or cardiac transplantation occurred within 3 years from RV-EMB in all patients.

Cardiac transplantation was performed after a median of 115 days (IQR 234) from RV-EMB, and death occurred after 153 days (500). 

Echocardiographic features at admission (e.g., reduced left ventricular function, shortening fraction, LVEDD and LVES) were associated with mortality/transplantation on bivariate analysis (LV-EF% *p* = 0.05; LV-SF% *p* = 0.01; LVEDD z-score *p* = 0.001; LVES z-score *p* = 0.001) as well as most of the hemodynamic parameters (LVEDP *p* = 0.08; LAP *p* = 0.02; mPAP *p* = 0.03; PVR *p* = 0.05; ScvO2 *p* = 0.02). Regarding biomarkers, NT-pro-BNP correlated significantly with a negative outcome (*p* = 0.02) while troponin showed no correlation. Worst clinical presentation with cardiopulmonary decompensation (*p* = 0.01), respiratory insufficiency (*p* < 0.0001), or hepatomegaly (*p* = 0.001) was related to worst outcome. No correlations were found with age, weight and length, or intra/peri-procedural course. 

**Figure 2:**
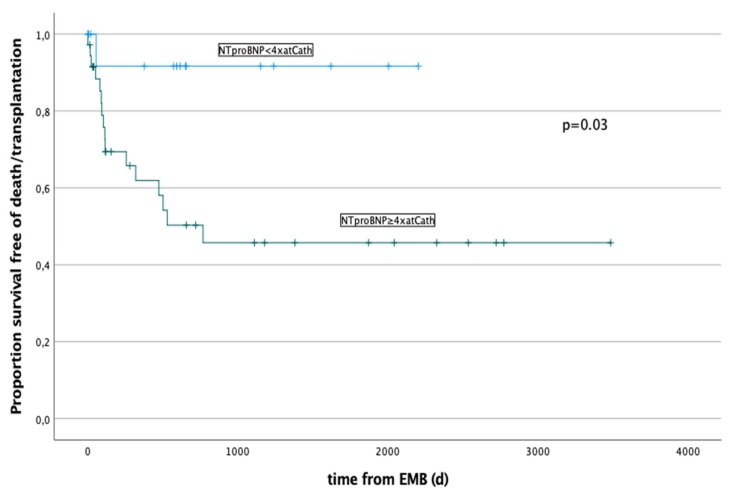
Survival function in relation to the maximal level of NT-pro-BNP prior to RV-EMB. Patients with a NT-pro-BNP ≥ 4× the normal value have a significantly increased risk (*p* = 0.03) for a negative outcome: death or cardiac transplant occurred in a total of 13/27 (48%).

In a multivariate risk analysis LV-EF% (*p* = 0.002, Hazard Ratio 0.94, C.I. 0.91–0.97), LVEDD z-score (*p* = 0.001; Hazard Ratio 1.3, C.I. 1.14–1.62), mPAP (*p* = 0.003; Hazard Ratio 1.05, C.I. 1.002–1.115), NT-pro-BNP (*p* = 0.004; Hazard Ratio 1.0, C.I. 1.003–1.0), and ScvO2 (*p* = 0.03; Hazard Ratio 0.93, C.I. 0.89–0.97) remained independently significant. The survival function in relation to the maximal value of NT-pro-BNP are showed in Figure 2.

## 4. Discussion

This study evaluated periprocedural course, hemodynamics, histological findings, and clinical outcome of RV-EMB in paediatric patients with suspected CMP or myocarditis. There are only few reports in literature focusing on this specific cohort of patients [16,18,27], since most studies report on results after RV-EMB performed in patients after cardiac transplantation [28,29]. 

### 4.1. Periprocedural Anaesthesiologic Management 

In our cohort we have shown that even if inotropic support might become necessary during performance of general anaesthesia and periprocedural management for RV-EMB in this specific patient group, only in few cases inotropic support needs to be increased due to the procedure. In fact, the number of patients receiving continuous inotropic support is high (50%) in our population, but only in 29% of the cases inotropic support had to be increased peri-procedurally. Pophal et al. [16] described a lower number of patients (4%), who received intravenous inotropic support, but only a small proportion of patients were evaluated for suspected myocarditis/CMP. A possible explanation to this difference is that our invasive assessment with RV-EMB was performed in median less than one week after hospital admission and prior to clinical re-compensation with circulatory support measures such as mechanical devices and eventual immunomodulatory or immunosuppressive therapy.

Multivariate analysis in their study demonstrated that the greatest risk of myocardial perforation and cardiac tamponade occurred in children being evaluated for possible myocarditis and requiring inotropic support. They concluded that the risk of endomyocardial biopsy is therefore highest in children on inotropic support. Although inotropic support was needed in 42/85 cases of our study, we have not found any association with serious periprocedural complications in this subpopulation of patients. Therefore, we think that RV-EMB can be performed also in patients under inotropic support early after hospital admission.

### 4.2. Complications during CC and RV-EMB

The timing of EBM remains a weighing between severity of cardiac failure, risk of periprocedural complications, and diagnostic impact [16,19,30]. The rate of severe complications of RV-EMB in children with suspected CMP or myocarditis is relatively low (2.9–5.2%) [27,31] and the incidence of RV-EMB related adverse events has been reported around 9.7–15.5% [16,18,27]. On the other hand, the rate of severe complications in an adult population is lower (0.15%), with an incidence of major complications around 0.20% [32]. 

Pophal et al. reported the largest series of 1000 RV-EMBs in children with an increased risk for complications with one biopsy related death secondary to cardiac perforation in a two-week infant with DCMP [16]. Cardiac perforation occurred predominately in children evaluated for myocarditis (5.2%) compared to post heart transplant patients (0.1%) [16]. The structurally altered myocardium due to the acute inflammation may be a reason for the greater perforation rate in this population [27]. Further risk factors were found to be younger patient age, smaller size, non-elective status, inotropic support, and femoral approach [16]. In a recent multicentre study on 206 paediatric RV-EMB an overall incidence of complication (minor and major) was reported as high as 9.7%, with a total of 6 (2.9%) ventricular perforations. Mortality rate was zero. In this study, biopsy during the first year of life and under inotropic support were associated with higher complications rates [27]. In a further multicentre study, the overall incidence of RV-EMB-related complications was 15.5% (31.2% in infants, and 6.8% in children), with an overall incidence of major complications of 11% [18]. Cardiac perforations only occurred in children below 1 year of age [18]. Technical and procedural limitations as for example inadequate biopsy forceps dimension for this age group might also play a role and complicate the procedure. 

In our cohort, we had two cases of cardiac perforation and one further major complication due to an electric conduction disturbance. On the other hand, and in contrast with other studies, no differences regarding age or inotropic support during the procedure could be described [16,18,27]. Of note is also, that the procedural-related complications were not associated with a longer hospital stay in our cohort. 

### 4.3. Histological and Serological Findings

Histology of EBM revealed acute, healing, or chronic myocarditis in nearly half of all cases, although the number of initially clinically suspected myocarditis cases was significantly lower (9 of 85 RV-EMBs). These findings underline the importance of RV-EMB in the diagnostic workup of children with clinically suspected myocarditis, as poor ventricular function seems to be caused by myocarditis more often than initially suspected by the clinician. This observation is contrary to earlier descriptions in the literature; in fact, Webber et al. (1994) [19] found that only 20% of all suspected children have proven histological myocarditis but without conducting immunohistological staining for the identification of immune cells in the heart. Recent improvements on RV-EMB analyses including immunohistology may account for the higher number of reported cases with signs of myocarditis in this study [13,24]. 

Histological diagnosis of myocarditis may influence further individual patient management because myocardial dysfunction may potentially be completely reversible [3,4,6,19,33]. In our cohort, all patients with an acute myocarditis survived without cardiac transplantation. This highlights the importance of a rapid, specific and, if necessary, also invasive therapy (e.g., ECMO or assist devices) in these cases. 

Importantly, histological examination together with PCR in heart biopsy specimens allows an etiological diagnosis in most cases [18]. Histopathological, immunohistological and virological information provided by RV-EMB have been used to try causal treatment strategies (immunosuppressive or antiviral therapies) [15]. However, results in literature are heterogeneous [14]. This may also be due to the different localisation of the biopsy in the right ventricle, stage of disease, genetic background, and variations in the definition of inflammatory cardiomyopathy [14]. A further complication is that even if a myocarditis is a frequent diagnosis in the paediatric population, it remains a rare diagnosis in the adult population, in which most of the causal treatment strategies are tested [15].

Regarding the impact of RV-EMB findings on specific medical treatment (e.g., immunosuppression) one must consider that some of these findings may also represent viral re-activation/persistence in myocardial cells or lymphocytes, rather than acute (systemic) viral infection. This is illustrated by the high amount of HHV6/7 in our study and from the fact that only five out of nine patients with histological acute myocarditis had a positive viral load in RV-EMB. 

The survival function in relation to the most frequent histological findings showed no significant difference among the survival of the groups with different diagnoses. Of note is the favourable clinical course of the patients with an acute myocarditis. On the other hand, almost half of the patients with a chronic myocarditis, dilated, restrictive and toxic CMP had an unfavourable outcome resulting in death or necessity for heart transplantation within three years from RV-EMB. 

### 4.4. Outcome and Outcome’s Predictors

One of the objectives of this study was to determine predictors of poor outcomes, defined as requirement for cardiac transplantation or death. In our cohort 18% received cardiac transplant and 13% of patients died [18]. Heart transplantation should rather be postponed in those cases of myocarditis in contrast to patients where genetic CMP is diagnosed [34]. Interestingly, there seems to be higher post-transplantation mortality in those children transplanted due to myocarditis associated terminal heart failure compared to non-myocarditis CMP [35]. A possible explanation could be that patients with a terminal heart failure due to CMP had a slow unset of the disease and a possibly favourable adaptation of the cardiovascular system that can overcome the early phase after heart transplantation with less difficulties. Both elevated LVEDP and mPAP as well as reduced LV-EF% and increased LVEDD z-score at admission predicted mortality/transplantation on multivariate risk analysis. Other studies had conveyed that the prognosis for patients with acute myocarditis depends on LV-EF% at presentation [5,36,37,38]. The independent role of high pulmonary pressure was also confirmed by other studies [37]. 

Troponin in the paediatric population is a marker that displays cardiac injury quickly and accurately also in myocarditis [39]; however, no correlation between outcome and troponin level was found [36]. Similar to our patient cohort, several other studies found an association of higher levels of BNP/NT-pro-BNP and poor outcome. One reason could be that, in contrast to cardiac troponins that are released due to cell wall compromise, BNP is synthesized in healthy cardiac myocytes from its precursor NT-pro-BNP [5,36,40,41,42,43]. Advanced imaging techniques such as strain analysis and new cardiac biomarkers, especially those related to extracellular matrix, have been appointed as early markers for poor outcome in patients after childhood cancer and could be considered for further analyses in this patients’ population [44]. 

### 4.5. Limitations

This study bares all limitations of a retrospective analysis. Further, other parameters such as cardiac MRI or genetic results were not evaluated, as well as an RV-EMB is rarely performed in patients below 1 year of age due to technical difficulties. The long study period is a strength of the study; however, clinical approaches changed over the last 20 years. In the future, larger paediatric studies with larger paediatric patient populations are needed and should especially evaluate the clinical impact of right ventricular endomyocardial biopsy results on further specific treatment and management of these patients during follow-up [37]. Lastly, the specific impact of the immunosuppressive/immunomodulatory treatments is difficult to determine because we did not perform any RV-EMB after treatment. 

## 5. Conclusions

Hemodynamic invasive data and histological/immunohistological findings are important for the definite diagnosis of myocarditis and different entities of cardiomyopathies, helping to stratify further clinical management and to estimate individual prognosis. In fact, all patients with an acute myocarditis survived. LV-EF% on echocardiography, hemodynamic data, NT-pro-BNP, and histology diagnosis help to predict unfavourable outcome. These patients might benefit from an earlier mechanical circulatory support in order to allow survival until transplantation. 

## Figures and Tables

**Table 1:** Clinical and echocardiographic findings at hospital admission.

	Median (IQR)/*n* (%)
*Patients caractheristics*	81 (100)
Age (years)	6.8 (9.9)
Weight (kg)	20.0 (32.2)
Body surface area (BSA)	0.8 (0.8)
*Clinical symptoms*	6 (7)
Fatigue (*n*)	55 (65)
Tachypnoea (*n*)	46 (54)
Cardiac murmur (*n*)	43 (51)
Hepatomegaly (*n*)	39 (46)
Tachycardia (*n*)	34 (40)
Cardiopulmonary decompensation (*n*)	18 (21)
*Echocardiographic findings*	
LV-ejection fraction (EF) (%)	35 (28)
LV-SF of 20% (%)	20 (16.5)
LVEDD z-score	+2.61 (3.73)
LVES z-score	+4.30 (4.83)

**Table 2:** Hemodynamic findings and periprocedural complication during CC with RV-EMB. Abbreviations: LVEDP = leftend-diastolicpressure, LAP = leftatrial pressure, mPAP = meanpulmonary artery pressure, PVR = pulmonary vascular resistance, CI = cardiacindex, ScvO2 = venousoxygensaturation.

Complications	Median (IQR)/*n* (%)
- *Major*	3 (3)
Myocardial perforation	2 (2)
Temporary complete AV block	1 (1)
- *Minor*	6 (7)
**Hemodynamic findings**	
LVEDP (mmHg)	17 (9)
LAP (mmHg)	15 (10)
mPAP ≥ 25 mmHg (*n*)	33 (34)
PVR (Wood Units/m^2^)	1.83 (1.87)
ScvO2 (%)	70 (13)

**Table 3:** Molecular biological detection of cardiotropic viruses performed by nested PCR from right ventricular endomyocardial biopsy (RV-EMB) and peripheral lymphocytes. Only in 9 RV-EMB the same pathogen was found in the myocardium as well as in the lymphocytes. Regarding the relationship between histological finding of myocarditis and viral detection only 33% the RV-EMB were positive for virus.

**PCR from RV-EMB (*n* = 79)**	***n***	**%**
Positive - Adenovirus	16 1	20 1
- EBV, PVB19	1	1
- HHV6	7	9
- PVB19	6	8
- BVP19, HHV6	1	1
Negative	63	80
**PCR from peripheral lymphocytes (*n* = 77)**	***n***	**%**
Positive - Adenovirus	33 1	43 1
- CMV	3	4
- EBV	4	5
- EBV, HH6, HH7	1	1
- EBV, HH7	1	1
- Enterovirus	2	3
- HHV6	8	10
- HHV6/7	2	3
- HHV7	6	8
- PVB19, HHV6	1	1
Negative	44	57
**Positive PCR from RV-EMB in myocarditis cases (*n* = 42)**	***n*/*n***	**%**
Myocarditis	14/42	33
- Chronic	7/28	25
- Healing	2/5	40
- Acute	5/9	56
Same virus in RV-EMB and Lymphocytes	9/77	12

## Data Availability

All authors take responsibility for all aspects of the reliability and freedom from bias of the data presented and their discussed interpretation.
